# Tumor-Derived Exosomes: Hidden Players in PD-1/PD-L1 Resistance

**DOI:** 10.3390/cancers13184537

**Published:** 2021-09-10

**Authors:** Valentin Vautrot, Hafidha Bentayeb, Sébastien Causse, Carmen Garrido, Jessica Gobbo

**Affiliations:** 1 Research Center UMR 1231, Label Ligue Nationale Contre le Cancer and LipSTIC, INSERM, F-21000 Dijon, France; valentin.vautrot@u-bourgogne.fr (V.V.); Hafidha.Bentayeb@u-bourgogne.fr (H.B.); Sebastien.Causse@u-bourgogne.fr (S.C.); Carmen.Garrido-Fleury@u-bourgogne.fr (C.G.); 2 Unité de Formation et de Recherches Sciences de la Santé, University of Bourgogne Franche-Comté, F-21000 Dijon, France; 3 Centre Georges-François Leclerc, F-21079 Dijon, France; 4 Centre Georges-François Leclerc, Early Phase Unit INCa CLIP², Department of Oncology, F-21079 Dijon, France; 5 Clinical Investigation Center CIC1432, Module Plurithématique, INSERM, F-21079 Dijon, France

**Keywords:** immunotherapy, resistance, tumor-derived exosomes, immunosuppression

## Abstract

**Simple Summary:**

Immunotherapies such as anti-PD-1/PD-L1 have garnered increasing importance in cancer therapy, leading to substantial improvements in patient care and survival. However, a certain proportion of patients present tumors that resist these treatments. Exosomes, small vesicles secreted by almost every cell, including tumor cells, have proven to be key actors in this resistance. In this review, we describe the involvement of immune checkpoints and immune modulators in tumor-derived exosomes (TEXs) in the context of cancer. We will focus on the most promising proteins under scrutiny for use in combination with PD-1 blockade therapy in a clinical setting: PD-L1, CTLA-4, TIM-3, CD73/39, LAG-3, and TIGIT. Finally, we will discuss how they can change the game in immunotherapy, notably through their role in immunoresistance and how they can guide therapeutic decisions, as well as the current obstacles in the field.

**Abstract:**

Recently, immunotherapy has garnered increasing importance in cancer therapy, leading to substantial improvements in patient care and survival. By blocking the immune checkpoints—protein regulators of the immune system—immunotherapy prevents immune tolerance toward tumors and reactivates the immune system, prompting it to fight cancer cell growth and diffusion. A widespread strategy for this is the blockade of the interaction between PD-L1 and PD-1. However, while patients generally respond well to immunotherapy, a certain proportion of patients present tumors that resist these treatments. This portion can be very high in some cancers and hinders cancer curability. For this reason, current efforts are focusing on combining PD-1/PD-L1 immunotherapy with the targeting of other immune checkpoints to counter resistance and achieve better results. Exosomes, small vesicles secreted by almost any cell, including tumor cells, have proven to be key actors in this resistance. The exosomes released by tumor cells spread the immune-suppressive properties of the tumor throughout the tumor microenvironment and participate in establishing metastatic niches. In this review, we will describe immune checkpoints and immune modulators whose presence in tumor-derived exosomes (TEXs) has been established. We will focus on the most promising proteins under scrutiny for use in combination with PD-1 blockade therapy in a clinical setting, such as PD-L1, CTLA-4, TIM-3, CD73/39, LAG-3, and TIGIT. We will explore the immunosuppressive impact of these exosomal proteins on a variety of immune cells. Finally, we will discuss how they can change the game in immunotherapy and guide therapeutic decisions, as well as the current limits of this approach. Depending on the viewpoint, these exosomal proteins may either provide key missing information on tumor growth and resistance mechanisms or they may be the next big challenge to overcome in improving cancer treatment.

## 1. Introduction

In recent years, immunotherapy in cancer mainly consisted in using immune checkpoint inhibitors (ICIs), monoclonal antibodies that prevent immunosuppression by blocking the engagement of checkpoint molecules, thereby reinvigorating the antitumor immune response. The first FDA-approved ICI in a clinical setting was ipilimumab, targeting CTLA-4, used as part of the treatment for advanced melanoma [1]. Almost concomitantly, it was followed by the development of two important anti Programmed cell Death-1 (PD-1) ICIs, nivolumab and pembrolizumab, which disrupt another immune checkpoint, the PD-1/Programmed cell Death Ligand 1 (PD-L1) interaction. PD-L1 is known as an immune checkpoint molecule that interacts with PD-1 to inhibit immunosurveillance. It can be expressed directly on the surface of tumor cells and recognized by PD-1, which is expressed on the surface of cytotoxic T-cells, to induce immune tolerance [2]. The effectiveness of these anti-PD-1/PD-L1 treatments is such that these inhibitors are now approved for use in the treatment of various types of cancer (classical Hodgkin lymphoma, metastatic melanoma, metastatic NSCLC (Non-Small Cell Lung Cancer), clear cell RCC (Renal Cell Carcinoma), HNSCC (Head and Neck Squamous Cell Carcinoma), and urothelial cancer) [3]. However, the benefit for the overall population is still marginal, and there is a strong heterogeneity in terms of response. It has been shown that non-responding patients can show resistance directly during the initial treatment, called a primary resistance, or may develop resistance after an initial response to treatment, therefore an acquired resistance [4]. Tumoral immune resistance is very complex and involves different mechanisms, such as tumor mutational burden leading to insufficient tumor immunogenicity, irreversible T-cell exhaustion, MHC (Major Histocompatibility Complex) dysfunction, and an immunosuppressive tumor microenvironment (TME), notably through the recruitment of MDSCs (Myeloid Derived Suppressor Cells) [5,6,7,8,9].

Recent studies on extracellular vesicles (EVs) have demonstrated that tumor-derived exosomes (TEXs) are bioactive nanovesicles with a potential role in tumor progression and resistance to immunotherapy. In fact, it has recently been demonstrated that exosomes, such as cells, are capable of expressing immunomodulators on their surface, and therefore are also capable of influencing the anti-tumor immune response [10]. It seems important to take into account the involvement of exosomes in resistance to treatment, which is why, in this review, we discuss the current state of knowledge on the contribution of immunosuppressor molecules contained in exosomes, and their effects on modulation of the immune system in the context of cancer. More specifically, we focus on the relationship between TEXs and combinational therapies including anti-PD-1/PD-L1 treatments.

## 2. Exosomes

### 2.1. Overview

**Figure 1:**
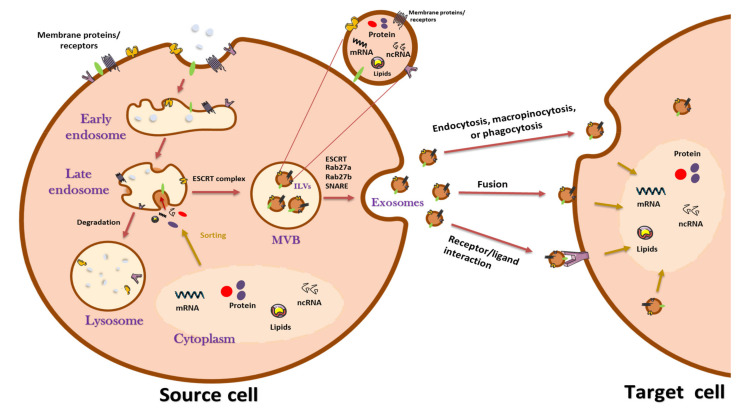
A schematic representation of exosome biogenesis and secretion. Exosomes are formed through the endocytic pathway. Invagination of the plasma membrane during endocytosis leads to the formation of early endosomes; exosome precursors called intraluminal vesicles (ILVs) are then formed by inward budding of late endosomes (cell RNAs, proteins, and lipid are incorporated at this step). The process of ILV biogenesis can be ESCRT-dependent or independent. Endosomes with an accumulation of ILVs are termed multivesicular bodies (MVBs); the fusion of MVBs with the plasma membrane releases the ILVs into the extracellular space by exocytosis, and these ILVs therefore become exosomes. The fusion of MVBs with the plasma membrane requires several crucial factors, such as Rab GTPases and SNARE complexes. In some instances, depending on the function and content of MVBs, they may fuse with the cell membrane and release exosomes or fuse with lysosomes for content degradation. Exosomes can directly interact with receptors on the target cell, fuse with the plasma membrane of the target cell, or enter into the target cell by endocytosis, macropinocytosis, or phagocytosis.

Exosomes are nanometer-sized EVs that are likely actors in intercellular communication and play a role in tumor physiopathology. Exosomes were first described in 1981 as nanovesicles secreted by different cell types in vitro, with a lipid composition different from the plasma membrane, suggesting a different and more complex origin compared with simple membrane budding [11]. Exosomes can be secreted by almost all cell types, including immune, blood, neuronal, epithelial, and also cancer cells [12,13]. They contain proteins retained from their cell of origin through their biogenesis process. These proteins include ESCRT (Endosomal Sorting Complex Required for Transport) and their partners (ALIX and TSG101), proteins from the plasma membrane (such as tetraspanins or MHC proteins) or the cytosol, but they exclude proteins from the nucleus, mitochondria, endoplasmic reticulum, and the Golgi apparatus [14]. Some proteins are fundamental to exosome biogenesis, such as the tetraspanins CD9, CD63, CD81. These are used as markers to validate exosome enrichment after nanovesicle isolation. Coupled with the physical and chemical characteristics of exosomes, such as size (diameter 50–200 nm) and density (1.13–1.19 g/mL), these proteins can help discriminate exosomes from other EVs (microvesicles, ectosomes, and apoptotic vesicles) and extracellular particles (microparticles). Exosomes can ultimately enter the circulation and have been detected in a variety of biological fluids, such as blood, urine, saliva, or breast milk, but also in malignant effusions such as pleural fluid and ascites [15,16]. It has been shown that exosomes can also contain miRNA, messenger RNA, or even long non-coding RNAs, which participate significantly in the regulatory effect of exosomes. There are two main ways by which the bioactive cargo of exosomes can influence the metabolism of recipient cells (Figure 1). The first is direct interaction of exosomal surface proteins with receptors of the target cell. The second is internalization of the content, either after fusion with the plasma membrane of the target cell or by endocytosis, macropinocytosis, or phagocytosis [17,18] (Figure 1). Nowadays, exosomes are of great clinical interest, especially in oncology. Due to their biogenesis, it has been established that TEXs contain a protein and lipid composition similar to that of the cells that secreted them.

### 2.2. Biogenesis of Exosomes

The biogenesis of exosomes involves different steps (Figure 1). The first step is a membrane invagination, leading to endosome formation and generation of the exosome precursors, called intraluminal vesicles (ILVs), by inward budding of endosomes. The endosomes with accumulations of ILVs are termed multivesicular bodies (MVBs). Then, the fusion of MVBs with the plasma membrane releases the ILVs into the extracellular space by exocytosis, and those ILVs become exosomes. More precisely, it appears that the formation of exosomes does not occur from a single process but the conjunction of different independent mechanisms [19]. One of the crucial elements involved in capturing proteins from the cell surface or the Golgi apparatus is the ESCRT transport machinery (Figure 1). Cytosolic RNAs and other proteins can directly access the interior of the vesicles being formed and be retained there after the separation of the vesicle from the cytosol resulting in free vesicles in MVBs [20]. However, this mechanism is not the only one that exists. Indeed, even after the inhibition of all ESCRT complexes using shRNAs, all of the compartments of the endocytosis pathway remain intact, and ILVs and exosomes can still be generated [21]. To become de facto exosomes, the ILVs must be released into the extracellular space. This release requires the transport of MVBs to the plasma membrane and their fusion. This journey is initiated and controlled by proteins called Rab GTPases that are also found associated with the membranes of different cellular compartments. The Rabs control the intracellular trafficking of membrane-containing components, and notably the fusion of MVBs with the plasma membrane; this latter step seems to require the additional role of proteins from the SNARE (soluble NSF attachment protein receptors) machinery. These proteins are present at the cell membrane and are essential players in exocytosis [17,22]. The biogenesis of exosomes is complex and only partially understood. However, there is no doubt that due to the way they are produced, exosomes are circulating delivery vessels, and their cargo reflects in a certain way the parent cell from which they are derived.

### 2.3. Functions of Exosomes in Cancer Development and Immunity

#### 2.3.1. Exosomes in Cancer Development

Initially described as a process of cellular waste disposal [23], exosomes later became the subject of a vast field of research. Their main role is the transfer of information between cells of an organism. They participate more generally in cell and tissue homeostasis by modulating the viability, status, and function of the cells they contact, and they can notably mediate tissue repair [24]. Exosomes can also contribute to the development and progression of tumors, including the transformation of normal cells into malignant cells and angiogenesis [25]. Théry et al. reported the pro-tumoral effect of exosomes by inhibiting the expression of Rab27a in tumor cells, causing a 50% decrease in exosome secretion, which inhibited tumor growth in vivo [26]. Exosomes have an important influence in almost every aspect of tumor growth and progression by acting on the tumor cells themselves and also favoring metastatic niches through the circulatory system. TEXs are messengers of choice for tumor cells to favor tumor growth [27,28] by directly activating signaling pathways such as PI3K (phosphoinositide 3-kinase)/AKT or MAPK (mitogen-activated protein kinase)/ERK [29]. TEXs can also participate in tumor vascularization by carrying pro-angiogenic factors such as IL-8, VEGF, MMP-2, or miR-21-5p, and reprogramming endothelial cells [30,31,32,33]. Furthermore, exosomes have a preponderant role in metastasis and cancer resurgence, based on several aspects either in the TME or at distant sites. They can notably promote epithelial to mesenchymal transition (EMT). Cancer Stem Cells (CSCs)-derived TEXs contribute to generating migratory and invasive tumor phenotypes and promote vascular degradation at pre-metastatic sites, notably through remodeling of the extracellular matrix. This influence of exosomes plays out in several ways such as the carrying of matrix metallo-proteases, proteolytic enzymes involved in the degradation of the extracellular matrix, or integrins, whose expression is decisive in maintaining CSC phenotypes [34,35,36]. Metastatic tumor exosomes also carry EMT-inducing factors, such as vimentin, annexin 2, and casein kinase II, which are absent in their non-metastatic equivalents [37]. Stromal cell-derived exosomes can participate in the selection of CSCs and induction of stemness, as is the case with multipotent mesenchymal stem cells, progenitor cells frequently found associated with tumors [38].

#### 2.3.2. TEXs in Immunity

Zitvogel et al. described exosomes as “tumor associated antigens” and immunostimulatory agents [39]. They have alternatively been described as immunosuppressors that regulate the functions of immune cells in the TME by expressing immunosuppressive or pro-apoptotic molecules on their surface, such as Fas-ligand, PD-L1, and IL-10 [40]. TEXs decrease the proliferation of T-cells [41,42] and inhibit the cytotoxic function of NK cells [43]. Several studies have shown that TEXs stimulate the infiltration of macrophages at the tumor site and their polarization into a pro-tumoral M2-like or TAM (Tumor-Associated Macrophages) phenotype, thereby promoting tumor progression [44,45]. TEXs can also reduce Dendritic Cell (DC) proliferation, maturation, and functions [46]. TEXs have been shown to positively influence the expansion, survival, and immunosuppressive functions of MDSCs. In breast tumor models, TEXs enhance the immunosuppressive activity of MDSCs by notably boosting the production of suppressive molecules [47]. Moreover, TEX-activated-MDSCs were capable of polarizing monocytes toward an M2 phenotype, favoring the formation of a tumor-friendly microenvironment [48]. The immunosuppressive effects of exosomes involving the activation of MDSCs can occur through a membrane-bound form of HSP70 (Heat Shock Protein 70) present at the surface of exosomes. These TEX-bound HSP70s can bind Toll-like receptor 2 (TLR2) at the surface of MDSCs, activating the STAT-3 pathway, which results in the suppression of neighboring T lymphocytes [49,50,51,52]. Finally, it has recently been shown that TEXs contain immune checkpoint proteins, such as PD-L1, TIM-3, or CD73/CD39, which suggests a potential function in ICI resistance [53,54,55,56,57].

## 3. Exosomes and Immune Checkpoints

**Figure 2:**
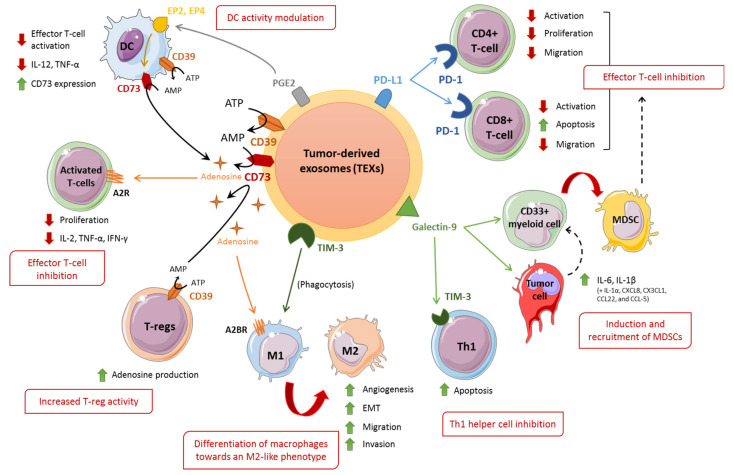
Potential immunosuppressive mechanisms of TEX in the context of immunotherapy resistance. TEX-bound CD73/CD39 can turn extracellular ATP into adenosine. Adenosine is a molecule possessing immunosuppressive properties in the TME. Adenosine binding to adenosine receptors (A2R, A2BR) on T-cells can directly inhibit T-cell activation. Adenosine also promotes differentiation of macrophages toward an M2-like phenotype. Prostaglandin E2 (PGE2)-containing TEX can also stimulate production of CD73 and subsequently adenosine by DCs, by activating PGE2 receptors (EP2, EP4). This results in an additional adenosine production. Moreover, TEX-bound TIM-3, upon phagocytosis by macrophages, can promote transition of the latter to a pro-tumoral M2-like phenotype. TEX-bound galectin-9 can promote apoptosis of T-helper cells (Th1) by binding to TIM-3 on the latter. TEX-bound galectin-9 can also promote the conversion of myeloid cells into tumor-favorable MDSCs by inducing the secretion of IL-6, IL-1β, and other cytokines by nasopharyngeal carcinoma cells and myeloid cells. Additionally, TEX-bound PD-L1 can inhibit activation, proliferation, and migration of effector T-cells such as CD4^+^ and CD8^+^.

In addition to CTLA-4 and PD-L1/PD-1, new ICIs have recently been investigated to overcome immunotherapy resistance. Current knowledge indicates that other checkpoint receptors (LAG-3, TIM-3, CD73/CD39 and TIGIT, OX40, CD40) complement the immune response regulation by PD-1, suggesting that their blockade combined with the use of anti-PD-1 antibodies could achieve better antitumor immune responses than anti-PD-1 therapy alone. For this reason, several studies have suggested combining immunotherapies, providing high hopes for improving the clinical efficacy of anti-PD-1 treatments. In this review, we will focus on the immune checkpoint elements, which have been extensively studied in a clinical setting and whose presence in exosomes has been described. The ClinicalTrials.gov, (accessed on 1 August 2021) registry references 467 studies where PD-1 inhibition is combined with other immunomodulators (https://clinicaltrials.gov/ct2/home, accessed on 1 August 2021). About 65% of these studies combine PD-1 with either PD-L1, CTLA-4, LAG-3, TIM-3, CD73/CD39, or TIGIT. In this review, we will explore the immunosuppressive impact of these exosomal proteins on a variety of immune cells. The spectrum of TEX actions is summarized in Figure 2 and Table 1.

### 3.1. Exosomes and PD-L1

PD-L1 (also known as B7-H1 or CD274) is a ligand of the PD-1 receptor that is encoded by the CD274 gene localized on chromosome 9p24.1 [80]. PD-L1 is expressed in activated T- and B-cells, DCs, monocytes, mesenchymal stem cells (MSCs), bone marrow-derived mast cells, and various immune-privileged organs [81]. PD-L1 expression is induced by pro-inflammatory cytokines such as IFN-γ and TNF [82], and regulated via the MAPK and PI3K/AKT pathways [83,84,85]. PD-L1 interacts with its receptor PD-1 on T-cells, modulating a series of processes linked to the T-cell-mediated immune response. It notably decreases their priming, proliferation, and functional maturation, and increases apoptosis. This pathway is critical for maintaining self-tolerance, preventing autoimmunity, and controlling T-cell responses to protect tissues from excessive inflammatory reactions [2,86]. In cancer, tumor cells use the PD-L1/PD1 pathway to escape from T-cell-mediated antitumor responses. PD-L1-overexpressing tumor cells can therefore survive, escape from surveillance by the immune system, and invade adjacent tissue [86,87].

Recently, clinical studies have demonstrated that PD-L1 is detected in exosomes purified from the plasma of patients (gastric, breast, pancreatic, oral-oesophageal cancers, HNSCC, NSCLC, and melanoma). It has also been found in exosomes from cell culture supernatants of various cancer cell lines (see Table 1) [56,60,61,64,66,71,88,89]. PD-L1 is expressed both at the surface of exosomes and within them. However, the mechanisms that control PD-L1 distribution among the different cellular compartments are not well understood [63,90]. ESCRT-accessory proteins seem to be involved in determining the cellular distribution of PD-L1. Deletion of the ESCRT complex and its accessory proteins (Rab27a, nSMase2, and Alix) decreases the release of PD-L1 through exosomes and increases PD-L1 levels at the cell membrane. In vitro and in vivo models have shown that PD-L1 can be transferred to other cells in a dose-dependent manner by exosomes [64]. Moreover, TEX-bound PD-L1 can inhibit T-cell-mediated immunity and enhance tumor growth in different tumor types, including lung [67], breast [64], prostate [65], head and neck [58,59], oral-oesophageal [71], and gastric cancers [69] (Figure 2). Moreover, it was shown that the presence of both PD-L1 and MHC-I was required for exosomes to exert their immunosuppressive activity [69]. These results may explain the higher immunosuppressive effect of exosomal PD-L1 compared with its soluble form [67].

TEX-bound PD-L1 can mimic the effect of PD-L1 at a cell membrane, i.e., inhibit T-cell activation and promote tumor progression (Figure 2). It was shown in different types of cancer (glioblastoma, HNSCC, melanoma, gastric, and lung cancer) that TEX-bound PD-L1 inhibited CD4^+^ and CD8^+^ T-cell activation and proliferation, resulting in anergic T-cells. This effect involved a decrease in secretion of interleukin 2 (IL-2), IFN-γ, and granzyme B [56,58,61,69,71]. In parallel, TEX-bound PD-L1 can induce apoptosis in CD8+ T-cells, and enhance the suppressing activity of T-regs in a dose-dependent manner [58]. Moreover, TEX-bound PD-L1 from HNSCC was able to reduce migration of CD4^+^ and CD8^+^ T-cells toward the tumor site, reducing the pressure of the immune response on tumoral cells [71].

Interestingly, TEX-bound PD-L1 from prostate cancer and melanoma were able to travel to the tumor’s draining lymph nodes and inhibit T-cell activation there, leading to T-cell exhaustion [54,65]. Moreover, the elimination of TEX-bound PD-L1 by inhibiting exosome secretion through Rab27 knockdown in a breast tumor model improved the efficiency of anti-PD-1 treatment and suppressed tumor growth [64]. Similar results were found in MC38 colon cancer and TRAMP-C2 prostate cancer mouse models [65]. These results indicate that TEX-bound PD-L1 can presumably inhibit the production and activation of T-cells at the source, even before their deployment in the TME. On the other hand, intravenous injection of exosomes containing PD-L1 enhanced tumor growth and decreased mouse survival in the MC38 model [65]. One of the possible hypotheses is that circulating TEX-bound PD-L1 could serve as decoys, diverting immunotherapy antibodies away from tumor cells, therefore resulting in resistance against anti-PD-L1 immunotherapy [91]. In fact, high levels of TEX-bound PD-L1 already indicate a higher probability of PD-1/PD-L1 blockade failure, as it usually indicates a far too advanced exhaustion of T-cells and insufficient TILs for recapacitation by treatment [65].

There has been little research into PD-1 containing exosomes. We found one report on the subject, which showed that in triple-negative breast cancer, exosomal PD-1 has an anti-PD-L1 function. This resulted in enhanced cytotoxic activity of T-cell and has potential as a therapeutic approach. The rationale would be to attenuate the immunosuppressive tumor microenvironment using membrane-bound immune checkpoint receptors that could negate the ligands [92].

Taken together, these data show that exosome-bound PD-L1 inhibits T-cells both in the tumor bed and at a distance from the tumor (lymph nodes, spleen) and, through these effects, contributes to tumor progression and resistance toward immunotherapy.

### 3.2. Exosomes and TIM-3

T-cell immunoglobulin and mucin domain 3 (TIM-3), first discovered in 2002 [93], is part of the TIM family of immunoregulatory proteins. It is encoded by the HAVCR2 (for hepatitis A virus cellular receptor 2) gene, located on the q13.2 region on chromosome 5. TIM-3 is expressed at the surface of various cells of the innate immunity, macrophages, DCs, monocytes, effector T-cells, NK, T-reg, and myeloid cells [94,95]. Its expression is widely associated with control of immune activity. The TIM-3 receptor is known to have an immunosuppressive effect upon binding with several known ligands (e.g., galectin-9 (gal-9), CEACAM1, or HMGB1). This interaction leads to T-cell inhibition by downregulating the TCR signaling pathway. Alternatively, it may directly disrupt the formation of immunological synapses during T-cell activation [96,97,98]. TIM-3 is a phenotypic marker of dramatic T-cell anergy, apoptosis, and exhaustion, in particular when found co-expressed with PD-1 in effector T-cells [94,99,100,101,102,103].

In the context of cancer, high TIM-3 expression can also be observed on tumor cells and sometimes concomitantly with its ligand gal-9 [104]. TIM-3 expression was observed in cancer cells (both primary and cell lines) sampled from lung [105,106], gastric [104] and cervical cancers [107], osteosarcoma [108], clear cell RCC [109], as well as melanoma [110] and leukemia stem cells [111,112]. The mechanisms underlying TIM-3 expression or the function of TIM-3 in non-immune cells are not well known yet. However, it was shown that suppressing TIM-3 expression inhibited the intrinsic invasive and migration properties of cervical carcinoma Hela cells [107]. This suggests that TIM-3 also participates in tumor growth in addition to its important role in immunosuppression. TIM-3 is co-expressed with several EMT markers in osteosarcoma cells [108]. It can promote tumor progression of the expressing cancer cell [109] but also of surrounding TIM-3 negative tumor cells. In line with these observations, higher TIM-3 expression in cancer cells was often correlated with higher metastasis rate, advanced cancer stages, and shorter overall survival [104,106,107].

TIM-3 and gal-9 have been found in TEXs from osteosarcoma, nasopharyngeal carcinoma (NPC), and NSCLC. In this regard, the presence of TEX-bound TIM-3 can participate in establishing a pro-tumoral environment by influencing cells of the TME, especially phagocytic cells (see Table 1 and Figure 2). TIM-3 containing TEXs secreted by the osteosarcoma cell line MG63 can undergo phagocytosis by macrophages. This induces polarization of the macrophage toward an M2-like phenotype, as indicated by the secretion of TGF-β, IL-10, and VEGF, and expression of the CD206, CD163, and Arg-1 markers [55] (Figure 2). This pro-invasive, pro-migration, and pro-EMT phenotype translated into an increased number of lung metastasis in vivo [55] (Figure 2). One of the hypotheses put forward is that phagocytosis of TIM-3-containing exosomes could increase the expression of TIM-3 in TAMs.

Among the TIM-3-activating ligands, only gal-9 was proven to be present and to have a role in TEXs. Gal-9 is a C-type lectin that can either remain inside the cell, be secreted, or be retained at the cell surface, often associated with other membrane proteins. There, it can interact with TIM-3 on T-helper type 1 (Th1) cells or be involved in the polarization of macrophages toward M2-like-tumor associated macrophages [95,103]. Accordingly, TEX-bound gal-9 has been detected in association with increased malignant features [72,73,74,75,113,114] (Table 1). In exosomes derived from the NPC cell lines (C15 and C17), TEX-bound gal-9 could promote apoptosis in Th1 helper cells (Figure 2). This effect could be abrogated with anti-TIM-3 and anti-galectin antibodies [73,74]. Moreover, exposure of NPC cells or CD33^+^ myeloid cells to TEX-bound gal-9 or other forms of gal-9 downregulates STING, a key adaptor protein in type I IFN signaling. This stimulates the production of several cytokines, in particular, IL-6 and IL-1β. These promote the differentiation of myeloid cells into tumor-favorable MDSCs and their recruitment to the TME [75]. Yet, the immunosuppressive function of TEX-bound gal-9 is still controversial. Indeed, some studies have reported that in situ overexpression of gal-9 was associated with more efficient immune functions, a more favorable clinical outcome, or a decrease in tumor migration and invasion, e.g., in breast cancer or gastric tumors [115,116,117]. This discrepancy could be explained by distinct activities of the intracellular and secreted forms of gal-9, which could further depend on the cellular context and tumor type. TEX-bound gal-9 may be an important factor in this equation.

Altogether, more studies on TEX-bound TIM-3 and TEX-bound gal-9 are needed to better comprehend the mechanisms of tumor immunoresistance and spreading. Studies are also required to evaluate their potential as therapeutic targets. This lack of information is also true for the other TIM-3 protein ligands, CEACAM1 and HMGB1.

### 3.3. Exosomes and CD73/CD39

CD73 and CD39 are enzymatic surface markers expressed from the ENTPD-1 (ectonucleoside triphosphate diphosphohydrolase 1) and NT5E (5′ nucleosidase ecto) genes, respectively. CD39 and CD73 can be found at the surface of T reg, Th17 helper, T-cells, neutrophils, macrophages, B-cells [118,119], and DCs [79,120]. They appear to be critical for the immunosuppressive activity of T-cells. These enzymes act sequentially to hydrolyze extracellular ATP. ATP is first hydrolyzed into AMP by CD39; AMP is then hydrolyzed by CD73, leading to the production of the small effector molecule adenosine. Adenosine is a purinergic mediator that, along with extracellular ATP, is produced in response to cellular stress, e.g., metabolic stress, tissue injury, and other types of injury, to maintain immune homeostasis [121,122,123]. Adenosine in particular generally acts as a brake on immune activity. The balance between ATP, AMP, and adenosine in the extracellular space is important for the regulation of tumor development and immune-escape mechanisms [124]. Adenosine can interact with numerous receptors from the adenosine receptor (AR) family (A1R, A2AR, A2BR, and A3R), which are present at the surface of various immune cells (DCs, macrophages, NK cells, T- or B-cells, and regulatory T-cells) [78,119,120,125,126].

Expression of AR and the ectonucleosidases in cancers has been described for both CD73 (e.g., CRC, prostate, breast, HNSCC, ovarian, and melanoma) and CD39 (e.g., melanoma, ovarian, head, and neck cancer) [127]. Tumor cells often show upregulation of CD73/CD39 expression, generally increasing adenosine concentrations in the TME, which in turn leads to increased tumor cell proliferation and invasion [128,129]. This upregulation of CD73/CD39 is caused by the specific conditions encountered in a tumoral context, such as high TGF-β concentration or hypoxia [130,131]. CD73 and CD39 are notably upregulated in a specific type of regulatory lymphocytes, referred to as peripheral T-regs, transformed by exposure to tumor antigens and tumor-derived factors [79,132]. They produce large amounts of adenosine and contribute to the reduction of anti-tumoral activity [79,133]. AR activation contributes to tumor growth and formation of metastases [125,134,135,136,137], notably by increasing T-cell anergy or promoting T-regs [138,139]. Interestingly, triggering AR can result in an increased expression of immune checkpoints such as CTLA-4, PD-1, LAG-3, and TIM-3 on CD8^+^ T-cells [140]. Several pre-clinical studies have demonstrated the synergistic effect of anti-PD-1 or anti-PD-L1 treatments in combination with the use of anti-CD73 antibodies. Numerous early-phase clinical trials testing this strategy have been completed or are still ongoing (e.g., in NCT02655822, NCT02503774, and many others).

Exosomes naturally bear CD73 and CD39, and the proteins are present in exosomes isolated from the plasma or pleural fluid of cancer patients (bladder, breast, prostate cancers, mesothelioma, HNSCC, and adenocarcinoma) [76,78,79]. As expected, TEX-bound CD73/CD39 is able to convert ATP to adenosine. TEX-bound CD73/CD39 from mesothelioma cells have been shown to inhibit T-cell activation via the binding of adenosine to the A2R receptor [76] (Figure 2). Moreover, TEX-bound CD73 was also able to amplify adenosine production in immune cells with low or no expression of CD73. For instance, TEX-bound CD73 in conjunction with CD39 on T-regs and DCs increase the conversion of ATP to adenosine [59,79]. In parallel, it has been shown that exosomes expressing Prostaglandin E2 (PGE2) can interact with PGE2 receptors (EP2, EP4) on DCs and lead to production of CD73 by the latter, thereby increasing adenosine production [77] (Figure 2). This adenosine production also decreases TNF-α and IL-12 secretion in DCs, blunting their pro-immunity properties. TEX-bound CD73/CD39 can also act on macrophages (Figure 2). Indeed, it has been reported in HNSCC that adenosine produced by TEX-bound CD73/CD39 promote the A2BR-mediated polarization of macrophages toward a pro-tumoral M2-like phenotype, which leads to an increase in production of IL-10, Arg-1, and angiogenesis factors (angiopoietin-1, endothelin-1, IL-8 and platelet factor 4) [78]. Finally, adenosine receptors seem to control exosome production [141]. All these studies suggest, at the very least, the replication of cellular CD73/CD39 activity on exosomes and their importance in adenosine production and activity.

### 3.4. What about CTLA-4, LAG-3, and TIGIT in TEX?

For some immune checkpoints currently under scrutiny, the involvement of exosomes has been demonstrated, and this knowledge has contributed to recent progress at a clinical level, particularly in the prediction of response to immunotherapy. For others, while the functional importance of exosomes has been hinted at, available information is sparse or sometimes non-existent. Indeed, although we have found several clinical trials testing anti-PD-1/PD-L1 in combination with anti CTLA-4, LAG-3, or TIGIT, the involvement of exosomes has not been really explored. One of the first attempts at immunotherapy was the association of an anti-CTLA-4 treatment with an anti-PD-L1 treatment. CTLA-4 is an immune checkpoint protein expressed at the surface of effector T-cells. Unfortunately, very little is known about the status of CTLA-4 in exosomes. However, higher levels of TEX-bound CTLA-4 and other immunosuppressive markers (PD-L1, COX2, CTLA-4, CD15s, or CD44v3) in the plasma of HNSCC patients have been shown to be associated with increased apoptosis of activated CD8^+^ T-cells [142,143].

Other immune checkpoints are currently being studied as potential targets in combination with anti-PD-1/PD-L1 treatments. Currently, there is a paucity of data on the transfer and expression of LAG-3 in exosomes. LAG-3 is an inhibitory receptor that binds to MHC-II [144]. It has been proposed that MHC-II may regulate LAG-3 levels in the TME, and consequently could be a potential biomarker for anti-LAG-3 therapies [145]. Several studies have reported that MHC-II is expressed in exosomes [146] and that MHC-II-containing exosomes derived from MHC-II+ tumor cells have immunostimulant effects [147]. However, validation of these mechanisms is required to verify whether exosomal MHC-II is involved in the escape from anti-LAG-3 therapy.

Finally, TIGIT is a co-inhibitory receptor mainly expressed by activated and regulatory T-cells and NK cells [148]. The role of TIGIT in tumor immune surveillance is analogous to the PD-1/PD-L1 axis in tumor immunosuppression. Inhibiting this signalization may improve the efficacy of immunotherapy, and anti-TIGIT therapies showed synergistic effects with anti-PD-1/PD-L1 [149]. Unfortunately, no evidence of a link between TIGIT expression and exosomes has been produced so far, and there is no exosome-related evidence either concerning its ligands on APCs or tumor cells, CD155, and CD112. Clearly, given the importance of these actors in the current search for new cancer therapies, especially in combination with anti-PD-1/PD-L1 strategies, there is a crucial need for information regarding their presence on exosomes and their impact on resistance to therapy.

## 4. Challenges and Future Directions

This review highlights and summarizes the current knowledge regarding the role of TEXs containing immune checkpoint molecules in the resistance to anti-PD-1/PD-L1 therapy in the treatment of cancer. TEX-bound PD-L1, TIM3, and CD73/CD39 are described as alternative signalizations pathways used by tumor cells to escape anti-PD-1 therapy. Even though there is no doubt about the involvement of exosomes in immunosuppression, many questions remain unanswered.

First, it seems obvious that the different checkpoints presented here can be co-expressed in TEXs, but few studies, if any, have investigated this. Taking into account all of the immunomodulators in the same samples would make it possible to better understand the mechanism of resistance put in place against anti-PD-1/PD-L1 treatment and optimize therapeutic strategies.

Secondly, although in the studies carried out in cancer cell lines, there is no doubt as to the origin of the exosomes, those isolated from cancer patients’ plasma can come from both malignant and non-malignant cells. For example, PD-L1 has also been described in immune cells [150], mesenchymal stem cells [151], or other cells in or outside of the TME [152,153]. Moreover, LAG-3, TIM-3, and TIGIT can be expressed in NK or CD8^+^ T cell [154]. Their expression in exosomes derived from cells other than the tumor cells is thus far not known. To solve this problem, recent techniques for the isolation and separation of exosomes, including size-, charge-, and affinity-based techniques have emerged [155]. Immunoaffinity methods appear to be a reliable isolation and discriminating technique for separating subpopulations of exosomes from human plasma [156]. It is clear that the phenotypic and functional evaluation of TEXs and exosomes derived from various non-malignant cells remains necessary in order to gain insight into their respective potential to induce changes in immune cells.

**Figure 3:**
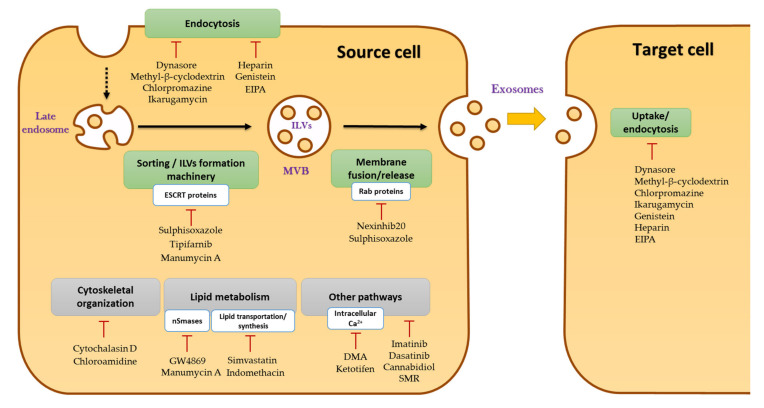
Cellular processes targeted by drugs inhibiting exosome formation/uptake. Cellular processes involved in exosomal biogenesis or uptake targeted by inhibitors are indicated in green inserts, at times accompanied by the specific components that are being targeted, in smaller inserts. Targeted processes affecting cells generally are indicated in grey inserts. **Uptake/Endocytosis:** Dynasore, Ikarugamycin, and Chlorpromazine target clathrin dependent endocytosis. Methyl-β-cyclodextrin removes the cholesterol from lipid rafts, and affects essentially caveolin-dependent endocytosis, but also clathrin-dependent endocytosis and macropinocytosis. Heparin inhibits cancer cell surface receptors, which depend on heparin sulfate proteoglycan co-receptors for the uptake of exosomes. Genistein is a tyrosine kinase inhibitor that indirectly interferes with the action of actin and dynamin on the plasma membrane necessary for endocytosis. EIPA can inhibit macropinocytosis. **Sorting/ILVs formation:** Sulphisoxazole, Tipifarnib, and Manumycin A target signaling pathways leading to depletion of ESCRT-dependent components. **Membrane fusion:** Nexinhib20 and Sulphisoxazole prevent the fusion of MVBs with the plasma membrane and subsequent exosome release, by inhibiting Rab or other proteins. **Cytoskeletal organization** is important for the membrane topology rearrangements necessary for vesicle formation, trafficking, secretion, and endocytosis. Cytochalasin D inhibits actin polymerization, which prevents trafficking of MVBs toward the plasma membrane, and may also inhibit macropinocytosis. Chloroamidine prevents the post-translational deamination of actin by the protein PAD (peptidylarginine deiminase), which is required for exosome release and uptake. **Lipid metabolism** is vital for exosome biogenesis and endocytosis. GW4869 and Manumycin A are selective inhibitors of nSMase2 (neutral sphingomyelinase 2), thereby blocking ceramide-mediated exosome biogenesis. Indomethacin reduces expression of the ABCA3 protein involved in lipid transport. Simvastatin, a cholesterol-lowering drug, decreases levels of the exosomal proteins ALIX, CD63, and CD81. **Other pathways:** Ketotifen (an antihistamine) and Dimethyl Amiloride (DMA) target intracellular calcium levels, important for regulating exosome release. Imatinib and Dasatinib are inhibitors of tyrosine kinases, while Cannabidiol and SMR are peptides found to inhibit EV release in general (adapted from Hayatudin et al., 2021 [161]).

Third, targeting TEXs is complementary to targeting the surface of tumor cells, and not redundant, because of the specific mechanisms they mobilize and their involvement in resistance to treatments. Hence, an improved understanding of the overall immunosuppressive role of TEXs in an immunotherapeutic context is important. In order to improve the efficacy of ICIs, the elimination of circulating exosomes has emerged as a novel therapeutic strategy. Different approaches are being explored, such as suppressing their generation and secretion using chemical inhibitors [157,158] or genetic manipulation [159]. Many molecules have been considered for their inhibitory effect on exosome release, targeting one or several steps of exosome biogenesis or uptake (Figure 3) [160,161,162]. These inhibitors can block (i) endocytosis (e.g., Dynasore, Methyl-β-cyclodextrin, Chlorpromazine, Ikarugamycin, Heparin, Genistein, EIPA) [163,164,165,166,167,168]; (ii) the protein machinery necessary for ILV formation (e.g., Manumycin A, Tipifarnib, Sulphisoxazole) [157,158,169]; (iii) the lipid metabolism (e.g., GW4869, Indomethacin, Simvastatin, Manumycin A) [170,171,172]; (iv) MVB membrane fusion and exosome release (e.g., Nexinhib20, Sulphisoxazole) [160,173]; and (v) cytoskeletal organization, which is important for vesicle formation, trafficking, and secretion (e.g., Cytochalasin D, Chloramidine) [164,174]. The list is not exhaustive, and the mechanisms of action of these inhibitors are also not mutually exclusive. Finally, some molecules with a less direct link with exosome biogenesis have also been found to inhibit exosome secretion (e.g., cannabidiol, SMR, imatinib, dasatinib) [175,176,177], while others affect intracellular calcium levels, which is important for exosome biogenesis and release (e.g., DMA, Ketotifen) [50,178]. One important aspect has to be taken into consideration, however: As mentioned in this review, exosomes are ubiquitous and participate in a variety of physiological processes. They are an important part of normal cell physiology. Consequently, there may be limitations to the strategy of inhibiting exosome production in general, which future studies must take into consideration to prevent adverse effects in patients following such therapies. The problem is similar to that of other strategies of exosome removal, such as extracorporeal haemofiltration [91]. To circumvent this problem, a possible solution would be targeting protein markers that are specific of cancer cells, for example, the membrane bound form of HSP70, prevalent in TEXs and cancerous cells, but not in healthy cells and exosomes derived from the latter [50,51,179]. An aptamer (A8) was specifically designed for this purpose [51].

Finally, in immunotherapy, one of the major challenges is establishing predictive biomarkers to determine the benefit of these drugs. The inherent characteristics of exosomes make them ideal candidates as a circulating pool of biomarkers. In fact, a prospective study on melanoma showed that analyzing the levels of TEX-bound PD-L1 could be helpful in predicting treatment efficacy and clinical outcomes [56]. TEX-bound PD-L1 are also being explored as biomarkers in NSCLC, small cell lung cancers, gastric cancer, HNSCC, CRC, RCC, hepatocellular carcinoma, esophageal carcinoma, and melanoma, and results to date have shown that exosomal-PD-L1 was (i) significantly higher before treatment in the plasma of non-responding patients; and (ii) decreased during therapy, and this decrease is associated with a decreased tumor burden [68]. In addition, in NSCLC, low levels of exosomal PD-L1 before PD-1 treatment were associated with prolonged progression-free survival [68]. Exosomal-TIM-3/gal-9, and CD73/CD39 are also being investigated as immunotherapy biomarkers, respectively, in NSCLC or HNSCC patients, and were found to be associated with many indicators of tumor progression, i.e., an aggressive phenotype, higher tumor burden, advanced stages of cancer, and more frequent distant metastasis [59,72].

## 5. Conclusions

To conclude, there is still a long way to go before we can fully understand all the molecular mechanisms of TEXs and exosomes derived from non-malignant cells implicated in immune regulation and drug resistance. TEXs bearing immune checkpoint components have great potential in clinical applications as new targets for immunotherapy and new biomarkers in liquid biopsies.

## Figures and Tables

**Table 1:** Characteristics of the immunomodulating proteins from tumor-derived exosomes presented in this review. PD-L1: programmed death-ligand 1; HNSCC: head and neck squamous cell carcinoma; IL: interleukin; IFN: interferon; TNF: tumor necrosis factor; EMT: epithelio-mesenchymal transition; MDSC: myeloid derived suppressor cells, NSCLC: non-small cell lung cancer; CRC: colorectal cancer; NPC: nasopharyngeal carcinoma.

Exosomal Protein	Type of Cancer	Source of Exosomes	Target Protein	Potential Exosome-Related Functions in Cancer
PD-L1	HNSCC	Plasma [58,59,60]	PD-1	Decreases the secretion of IL-2 and IFN-γ Suppresses the proliferation of CD4^+^ T-cells Induces apoptosis in CD8^+^ T-cells Enhances the suppressor activity of T-reg cells
	Glioblastoma	Plasma; Primary cancer cells lines G34, G35, G44, G157 [61]		Inhibits CD4^+^ and CD8^+^ T-cell activation
	Breast	Cancer cell lines MDA-MB-231, HCC1954, SKBR3, EFM-192A [62,63,64]		Decreases the secretion of IL-2 Suppresses T-cell activation
	Prostate	Cancer cell lines DU145, PC3, LnCap, TRAMP-C2 [65]		Inhibits T-cell activation Causes T-cell exhaustion Reduces spleen size
	Melanoma	Plasma; Cancer cell lines SK-Mel-2, SK-Mel-28, Mel624 and WM9 (human) and B16F10 (murine) [54,56,65]		Reduces production of granzyme B, IFN-*γ*, IL-2, and TNF-*α* Inhibits proliferation of T-cells Reduces migration of CD4^+^ and CD8^+^ T-cells
	NSCLC	Serum; plasma; Cancer cell lines A549, H460, H1975 [66,67]		Decreases IFN-*γ* production in a dose-dependent manner
	Lung Squamous Cell Carcinoma and adenocarcinoma	Plasma [68]		Inhibits INF-γ production Promotes apoptosis of CD8^+^ T-cells Promotes tumor growth
	Gastric cancer	Plasma [69,70]		Reduces CD4^+^ and CD8^+^ T-cell numbers, and reduces granzyme B production Suppresses T-cell activation
	Oral-oesophageal	HNSCC cancer cell lines SCC90 (human) and SCCVII (murine) [71].		Reduces migration of CD4^+^ and CD8^+^ T-cells
TIM-3	Osteosarcoma	Cancer cell line MG63 [55]	(Non Available)	Promotes the pro-tumoral M2 phenotype in macrophages Increases invasion, migration, EMT of osteosarcoma cells; increases lung metastasis in in vivo nude mouse model
	NSCLC	Plasma [72]		Associates to malignancy and promotes metastasis
Gal-9	NPC	Plasma (patients) [73] Plasma from C15 and C17 xenografted mice [73]	TIM-3	Induces apoptosis in helper T-cells
		NPC xenografts cell lines (C15 and C17) supernatant [74]		Suggested to inhibit proliferation of peripheral blood resting T-cells
		Cancer cell lines TW03 [75]	(Non Available)	Promotes maturation into MDSCs from myeloid CD33^+^ cells
CD73/CD39	Bladder, breast, colorectal	Cancer cell lines HT1376 (bladder), CACO2 (CRC), MCF7 (breast) [76]	Adenosine Receptors	Produces adenosine (immunosuppressor)
	Prostate	Cancer cell lines DU145, PC3 [76,77]		Produces adenosine (immunosuppressor)
	Mesothelioma	Pleural effusion; custom cancer cell line (Meso) [76]		Reduces proliferation and activity (IL-2, TNF-α secretion) of activated T-cells
	HNSCC	Plasma; cancer cell line UMSCC47 [78]		Promotes A2BR-mediated polarization of macrophages toward a pro-tumoral M2-like phenotype, leading to increased angiogenesis
		Plasma [79]		Produces adenosine (immunosuppressor) Induces adenosine production by CD39^+^ T-regs
		Plasma [59]		Produces adenosine (immunosuppressor) especially at later cancer stages
